# Body Roundness Index Versus Body Mass Index: Differential Associations With Obstructive Sleep Apnea Syndrome and All‐Cause Mortality in US Adults Aged 20 Years and Older

**DOI:** 10.1002/brb3.71109

**Published:** 2025-12-02

**Authors:** Xiaodong Chen, Xianglian Chen, Hongwei Liu, Minheng Zhang

**Affiliations:** ^1^ Department of Otolaryngology, Taiyuan Central Hospital The Ninth Clinical Medical College of Shanxi Medical University Taiyuan Shanxi Province China; ^2^ Department of Neurology, Taiyuan Central Hospital The Ninth Clinical Medical College of Shanxi Medical University Taiyuan Shanxi Province China; ^3^ Department of Gerontology The First People's Hospital of Jinzhong Yuci Shanxi Province China

**Keywords:** all‐cause mortality, body mass index, body roundness index, NHANES, obstructive sleep apnea syndrome

## Abstract

**Background:**

Being obese considerably elevates the risk of both obstructive sleep apnea syndrome (OSAS) and mortality. Although body mass index (BMI) is a common measure, it neglects visceral adiposity, a critical factor in the development of cardiometabolic and respiratory dysfunction. By integrating waist circumference and height, the body roundness index (BRI) delivers a more accurate evaluation of central fat distribution, potentially improving the prediction of OSAS and mortality.

**Methods:**

We analyzed data from 13,854 US adults aged ≥20 years from the NHANES 2005–2008 and 2015–2018 cycles. Using multivariable logistic and Cox regression models, along with restricted cubic spline and subgroup analyses, the connections of BRI and BMI with OSAS and all‐cause mortality were examined, and ROC curves were employed to compare predictive performance.

**Results:**

Both BRI and BMI were significantly associated with increased OSAS risk, with stronger associations observed for BRI (adjusted odds ratio [OR] = 3.060, 95% confidence interval [CI]: 2.880–3.251, *p* < 0.001) than for BMI (adjusted OR = 1.573, 95% CI: 1.535–1.612, *p* < 0.001). In OSAS patients, BRI was positively associated with all‐cause mortality (adjusted hazard ratio [HR] = 1.087, 95% CI: 1.036–1.141, *p* < 0.001; Q4 vs. Q1: HR = 1.423, 95% CI: 1.073–1.887, *p* = 0.014), while BMI showed an inverse crude association that became marginally positive after adjustment (adjusted HR = 1.021, 95% CI: 1.001–1.040, *p* = 0.038; Q4 vs. Q1: HR = 1.378, 95% CI: 0.966–1.964, *p* = 0.037). BRI demonstrated superior predictive value for mortality (area under the curve [AUC] = 0.610) compared to BMI (AUC = 0.521; *p* < 0.001), despite comparable performance for OSAS prediction (BRI AUC = 0.793 vs. BMI AUC = 0.790; *p* = 0.236).

**Conclusion:**

BRI and BMI showed comparable ability to predict OSAS risk; however, BRI demonstrated superior value in mortality stratification, emphasizing its clinical significance for assessing visceral fat‐related prognosis. Because OSAS was identified using a validated questionnaire rather than polysomnography, minor classification errors may exist, warranting cautious interpretation of the findings.

## Graphical abstract

The analysis indicates differential associations of body roundness index and body mass index with obstructive sleep apnea and subsequent mortality, subtly pointing to the importance of adiposity distribution in shaping clinical trajectories.

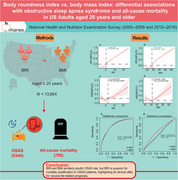

## 1 Introduction

Obesity is a well‐established determinant of both obstructive sleep apnea syndrome (OSAS) and all‐cause mortality. However, conventional anthropometric measures, most notably body mass index (BMI), provide limited insight into the distribution of body fat, particularly visceral adiposity, which has a disproportionately large impact on cardiometabolic and respiratory dysfunction (Jang et al. 2023; Gordon and Hsueh 2021; Strack et al. 2022; Javed et al. 2022; Sacramento et al. 2023). Although BMI is calculated as weight in kilograms divided by the square of height in meters and remains the prevailing clinical standard for defining obesity, it does not distinguish between fat and lean tissue or adequately capture central obesity, a major contributor to systemic inflammation and upper airway obstruction in individuals with OSAS (Romero‐Corral et al. 2008; Lee et al. 2012). Recent evidence has drawn attention to alternative indices such as the body roundness index (BRI), which incorporates waist circumference (WC) and height to geometrically model body shape and fat distribution with greater precision (Thomas et al. 2013). BRI has shown superior predictive utility due to its sensitivity to visceral fat accumulation, a phenotype strongly associated with insulin resistance, elevated blood pressure, and increased severity of OSAS, regardless of total body weight (Rico‐Martín et al. 2020). In view of BMI's diagnostic limitations, assessing the capacity of BRI to discriminate risk related to OSAS and mortality may offer more effective strategies for clinical decision‐making and public health interventions.

OSAS, characterized by recurrent upper airway collapse during sleep, affects approximately 34% of middle‐aged men and 17% of women in the United States, with obesity constituting the most prominent modifiable risk factor (Peppard et al. 2013). While BMI is routinely used to assess OSAS risk, accumulating evidence indicates that measures of central adiposity, such as WC and waist‐to‐hip ratio, exhibit stronger correlations with OSA severity. This association likely reflects the impact of fat deposition in the cervical and abdominal regions, which compromises airway patency and disrupts ventilatory control (Ma et al. 2022). In a prospective cohort study, Garzon et al. (2020) reported that WC was a more accurate predictor of incident OSA than BMI, underscoring the need for adiposity indices that more precisely capture central fat distribution (Garzon et al. 2023). The BRI, derived from WC and height, has been proposed as a superior proxy for visceral adiposity relative to BMI; yet, its utility in predicting OSAS remains underexplored (He et al. 2025). Furthermore, although both BRI and BMI have been linked to cardiovascular mortality, their differential associations with all‐cause mortality in individuals presenting with OSAS remain insufficiently characterized, pointing to a significant gap in current epidemiological understanding (Sahakyan et al. 2015).

All‐cause mortality risk among individuals with obesity is determined not only by the total volume of adiposity but also by its regional distribution, with visceral fat imparting substantially greater metabolic and pro‐inflammatory burdens than subcutaneous fat (Neeland et al. 2019). In a recent analysis of the National Health and Nutrition Examination Survey (NHANES) cohort, Chen et al. (2025) demonstrated that the BRI more accurately predicted cardiovascular mortality than the conventional BMI, highlighting its promise as a superior prognostic indicator. Although the association between OSAS and cardiovascular mortality is well established, no prior investigations have systematically compared the predictive performance of BRI and BMI in the context of OSA‐related mortality (Gami et al. 2013). This study seeks to address this evidentiary gap by analyzing nationally representative data from US adults aged 20 years and older to determine whether BRI exhibits stronger associations than BMI with OSAS symptomatology and all‐cause mortality. By clarifying the differential prognostic value of these adiposity measures, our findings aim to refine risk stratification strategies and support the integration of BRI into routine clinical assessment, particularly among individuals at heightened risk for sleep‐disordered breathing.

## 2 Methods

### 2.1 Study Participants

**FIGURE 1:**
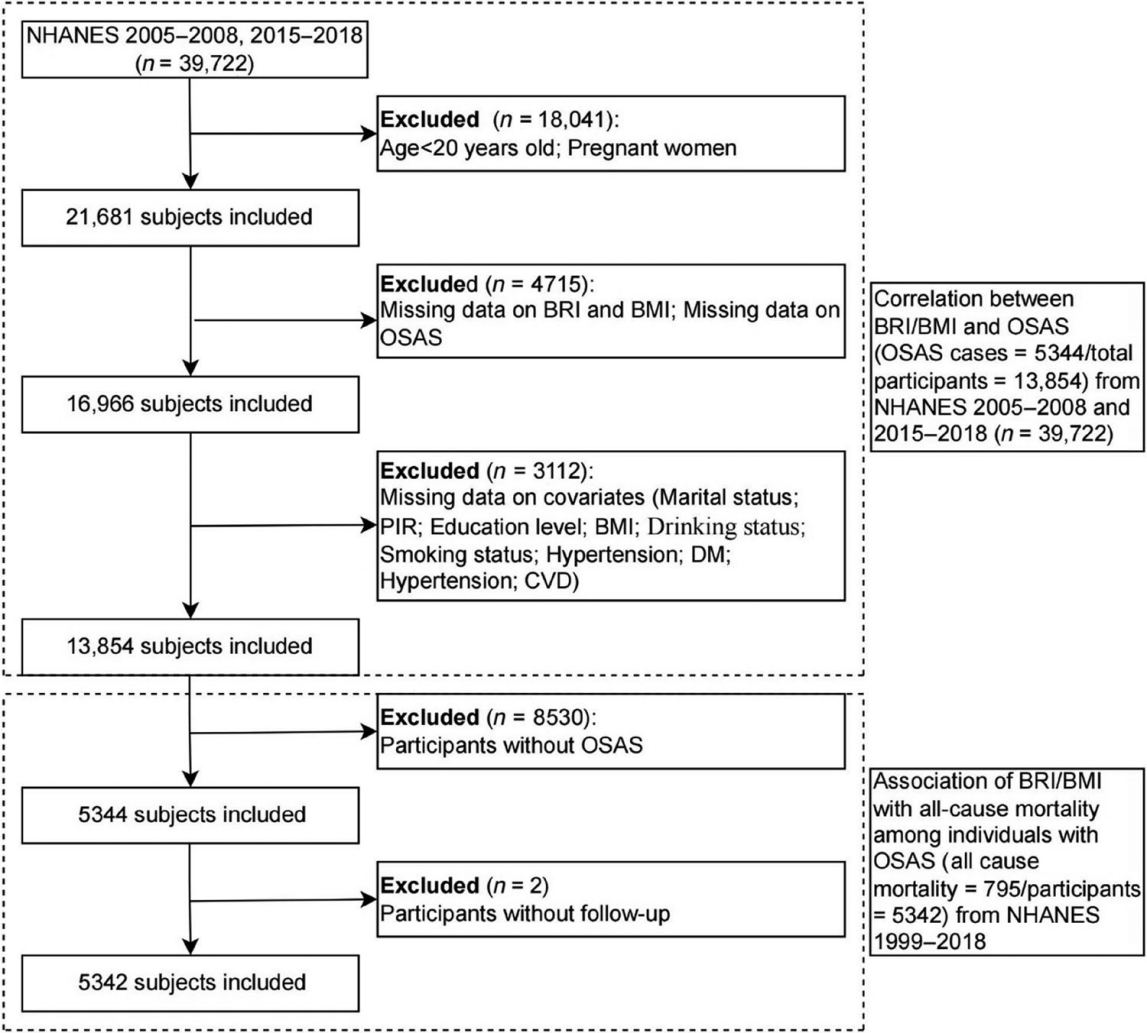
The flow diagram for selecting participants. BRI, body roundness index; BMI, body mass index; OSAS, obstructive sleep apnea syndrome; PIR, poverty‐to‐income ratio; DM, diabetes mellitus; CVD, cardiovascular disease; NHANES National Health and Nutrition Examination Survey.

This study utilized data from the NHANES, a nationally representative cross‐sectional survey of US adults conducted by the Centers for Disease Control and Prevention. We included participants aged ≥20 years with complete data on BRI, BMI, OSAS, and mortality status. Exclusion criteria were (1) pregnancy, (2) missing anthropometric or OSAS‐related data, and (3) missing data for any predefined covariates to maintain analytic consistency. A total of 13,854 participants who met the established eligibility standards were part of the final analytical cohort from the NHANES cycles of 2005–2008 and 2015–2018. Among the individuals, 5344 were clinically diagnosed with OSAS, and 795 cases of all‐cause mortality due to OSAS‐related complications were recorded during the follow‐up period (Figure 1). All participants provided written informed consent, and the NHANES protocols were approved by the NCHS Research Ethics Review Board. The analysis was considered exempt from extra ethical review due to its use of secondary analysis on de‐identified, publicly accessible data.

### 2.2 Definition of BRI and BMI

The Mobile Examination Center employed trained health technicians to collect physical measurements using standardized procedures. Prior to anthropometric measurements, all participants were directed to remove their shoes and heavy clothing. To maintain accuracy and participant comfort, trained professionals conducted the measurements in a private and comfortable environment. BRI is an innovative index for body shape evaluation. The index, derived from height (cm) and WC (cm), measures visceral fat content, where a higher number reflects more pronounced fat accumulation. BRI was computed by 364.2 − 365.5 × (1 − [WC (m)/2π]2 / [0.5 × height (m)]^2^)^½^ (Thomas et al. 2013). BMI is an internationally accepted measure used to evaluate body fat and determine if an individual's weight is considered healthy. The calculation involves dividing the weight in kilograms by the square of the height in meters (kg/m^2^).

### 2.3 Assessment of OSAS

To determine the risk of obstructive sleep apnea (OSA; range 0–1.0), we applied a modified multivariable apnea prediction index using data from NHANES (Maislin et al. 1995; Kariuki et al. 2022). The original model developed by Maislin et al. (1995) quantified OSA symptoms, including loud snoring, breathing pauses, and gasping or snorting during sleep, using a 5‐point Likert scale (0 = *Never*; 1 = *Rarely*; 2 = *Sometimes*; 3 = *Often*; 4 = *Always*, equivalent to five to seven times per week). In NHANES, four sleep‐related questionnaire items (“snoring,” “snorting or gasping,” and “stopping breathing during sleep”) were translated into a 0–4 scale, with higher values denoting more frequent events based on response frequency. The calculation of the mean did not include missing responses. The mean of the available symptom scores was combined with age, sex, and BMI in a logistic prediction equation adapted from Kariuki et al.’s (2022) model. The resulting index provided a continuous score from 0 to 1, which represents the predicted probability of OSA risk. Those with an index equal to or greater than 0.50 were categorized as high risk for OSAS, matching an Apnea–Hypopnea Index of 10 or more events per hour in validation cohorts (sensitivity 88%, 95% CI 84%–92%; specificity 55%, 95% CI 48%–62%) (Maislin et al. 1995; Kariuki et al. 2022).

### 2.4 Measurement of Covariates

According to NHANES protocols, the analysis was adjusted for a thorough set of covariates that influence both measures of adiposity and sleep‐related outcomes. Demographic variables included age (modeled continuously), sex (male or female), race or ethnicity (categorized as Mexican American, non‐Hispanic White, non‐Hispanic Black, other Hispanic, or other and multiracial), and marital status (classified as married or partnered versus single, divorced, or widowed). To assess socioeconomic status, educational attainment (less than high school, high school graduate, or postsecondary education) and the poverty–income ratio (PIR) were categorized into three groups (<1.3, 1.3–3.5, and ≥3.5). Behavioral covariates included smoking and drinking status, with individuals categorized by smoking history as never smokers (fewer than 100 cigarettes in a lifetime), former smokers (100 or more cigarettes but not currently smoking), or current smokers (100 or more cigarettes with ongoing use). Alcohol use was similarly classified as never, former, or current drinking, with heavy drinking defined as the consumption of four or more alcoholic beverages per day for women and five or more for men. Clinical covariates involved self‐reported conditions diagnosed by physicians, confirmed by medication use when it was available. Hypertension, according to the 2017 ACC/AHA guideline for adults, is defined as having an average systolic blood pressure of 130 mm Hg or above, a diastolic blood pressure of 80 mm Hg or above, or the current use of medication to lower blood pressure (Whelton et al. 2018). Diabetes mellitus (DM) was identified based on a physician's diagnosis reported by participants or confirmed through laboratory evidence when available, including fasting plasma glucose levels of at least 126 mg/dL (7.0 mmol/L), glycated hemoglobin (HbA1c) values of 6.5%, or current treatment with glucose‐lowering agents, in accordance with the diagnostic standards of the American Diabetes Association (ElSayed et al. 2023). Cardiovascular disease (CVD) was described as a self‐reported diagnosis by a physician of coronary heart disease, myocardial infarction, heart failure, angina, or stroke, consistent with previous NHANES definitions.

### 2.5 Assessment of All‐Cause Mortality

The study focused on all‐cause mortality among OSAS patients, which refers to death from any cause. The NHANES data were matched with the 2019 National Death Index data (https://www.cdc.gov/) to acquire mortality statistics, using personalized identifiers such as name, gender, and date of birth. The follow‐up duration was calculated from the date of the participant's initial interview, conducted by Centers for Disease Control and Prevention employees, to the date of death documented.

### 2.6 Statistical Analysis

Incorporating the complex sampling design of NHANES, all analyses employed the appropriate weights, strata, and primary sampling units. In line with NHANES analytical guidelines, combined survey weights were produced by dividing each 2‐year cycle weight by the total of four cycles and merging the datasets from 2005–2008 and 2015–2018. By using this approach, the estimates were ensured to be nationally representative of the US population over the study period (CDC/NCHS). Continuous variables were reported as weighted means plus or minus standard errors, while categorical variables were represented as weighted proportions. To analyze the associations between obesity indices, particularly the BRI and BMI, and the risk of OSAS, multivariable logistic regression models were utilized. Analysis of each index was performed as a continuous variable, standardized for a 1 standard deviation increase, and as a categorical variable using quartile distributions. The proportional hazards assumption for each Cox regression model was analyzed using Schoenfeld residuals and global tests. No substantial violations of the PH assumption were detected, showing that the models conformed to the proportionality criteria. The computation of variance inflation factors (VIFs) was done to check for multicollinearity, with all values being less than 2, suggesting no collinearity among the covariates (Tables S1 and S2). To guarantee transparency in the model and control for possible confounding variables, covariates were chosen based on existing epidemiological evidence and biological plausibility regarding their effects on adiposity and sleep‐related outcomes. Three models featuring hierarchical levels and incremental adjustments were constructed. Model 1 was left unadjusted, whereas Model 2 incorporated demographic and socioeconomic variables, including age, sex, race/ethnicity, marital status, education level, and PIR. In Model 3, further behavioral and clinical variables were added, including smoking status, drinking status, hypertension, DM, and cardiovascular disease. The stepwise approach of this modeling framework permitted us to investigate the incremental effects of covariates on the association between adiposity indices (BRI and BMI) and study outcomes. Results were presented as odds ratios (ORs) with corresponding 95% confidence intervals (CIs). In mortality analysis, Cox proportional hazards models calculated hazard ratios (HRs) with 95% CIs, using the follow‐up time from the NHANES examination until either death or censoring. Subgroup analyses were conducted to examine effect modification across clinically relevant subpopulations. Interaction terms between each stratification variable and BRI or BMI were included in fully adjusted logistic or Cox models, with statistical significance assessed via Wald tests. All models applied consistent covariate adjustments and accounted for NHANES's complex survey design using appropriate sampling weights and variance estimation. Using restricted cubic splines (RCSs) with three knots at the fifth, 35th, 65th, and 95th percentiles, nonlinear relationships were explored, and likelihood ratio tests were used to compare these with linear models. Kaplan–Meier estimation was applied in survival analyses to compare mortality risk among BRI/BMI quartiles (Q1–Q4), with follow‐up from the beginning to death or censoring. Mortality data were linked through the National Death Index using probabilistic matching, and survival differences were assessed with weighted log‐rank tests, which considered the complex survey design of NHANES. Receiver operating characteristic (ROC) curve analyses were extensively conducted to compare the predictive performance of BRI and BMI regarding OSAS risk and all‐cause mortality. We calculated area under the curve (AUC) values from multivariable‐adjusted logistic regression models for OSAS prediction, and for mortality, we used time‐dependent ROC methods to account for censored survival data. Using the survIDINRI package in R, we calculated the Integrated Discrimination Improvement (IDI) and the category‐free Net Reclassification Improvement (NRI) to assess the incremental prognostic value of BRI relative to BMI in predicting mortality risk among individuals with OSAS. By accounting for right‐censored survival data, these indices provide alternative assessments of the performance of model reclassification. Using 50 iterations of permutation‐based resampling, two‐sided *p* values were calculated to ensure robust inference. Analyses were carried out using R 4.4.1, where a two‐tailed *p* value of under 0.05 was used to establish statistical significance.

## 3 Results

### 3.1 Baseline Characteristics

A total of 13,854 participants from the NHANES 2005–2008 and 2015–2018 cycles were included in the analysis, of whom 5344 (38.6%) were diagnosed with OSAS, while 8510 did not meet the diagnostic criteria. Participants with OSAS were significantly older (mean age 53.5 ± 0.3 vs. 42.4 ± 0.4 years, *p* < 0.001), demonstrated higher levels of adiposity (BRI: 6.94 ± 0.06 vs. 4.45 ± 0.03; BMI: 33.8 ± 0.2 vs. 26.4 ± 0.1; both *p* < 0.001), and were predominantly male (76.1% vs. 35.2%, *p* < 0.001) (Table 1). Significant group differences were also observed in racial/ethnic distribution (non‐Hispanic White: 71.9% vs. 67.9%, *p* < 0.001), marital status (married: 74.6% vs. 63.9%, *p* < 0.001), and educational attainment (postsecondary education: 59.4% vs. 63.2%, *p* = 0.008) (Table 1). Additionally, the OSAS group exhibited markedly higher prevalence of comorbidities, including hypertension (54.7% vs. 25.9%), DM (24.0% vs. 7.2%), and cardiovascular disease (13.0% vs. 4.4%; all *p* < 0.001) (Table 1).

**TABLE 1:** Weighted baseline characteristics of participants categorized by OSAS status.

Characteristic	Total	No OSAS	OSAS	p value
	*N* = 13,854	*N* = 11,027	*N* = 5344	
Age, year, mean (SE)	46.340 (0.339)	42.382 (0.376)	53.511 (0.339)	<0.001
BRI, mean (SE)	5.337 (0.045)	4.453 (0.034)	6.938 (0.061)	<0.001
BMI, mean (SE)	29.027 (0.125)	26.391 (0.089)	33.803 (0.165)	<0.001
Gender, *n* (%)				<0.001
Female	6763 (50.244)	5497 (64.778)	1266 (23.913)	
Male	7091 (49.756)	3013 (35.222)	4078 (76.087)	
Race, *n* (%)				<0.001
Mexican American	2250 (7.937)	1400 (8.090)	850 (7.661)	
Non‐Hispanic Black	2946 (10.486)	1770 (10.569)	1176 (10.336)	
Non‐Hispanic White	5982 (69.302)	3544 (67.902)	2438 (71.837)	
Others	2676 (12.275)	1796 (13.439)	880 (10.166)	
Marital status, *n* (%)				<0.001
Never married	2328 (16.599)	1846 (20.926)	482 (8.761)	
Divorced/separated/widowed	2637 (15.700)	1568 (15.176)	1069 (16.649)	
Married/living with partner	8889 (67.701)	5096 (63.898)	3793 (74.590)	
Education level, *n* (%)				0.008
High school	3234 (14.091)	1885 (13.759)	1349 (14.692)	
Less than high school	3231 (24.084)	1946 (23.049)	1285 (25.958)	
More than high school	7389 (61.825)	4679 (63.192)	2710 (59.350)	
PIR, *n* (%)				<0.001
<1.3	3828 (18.094)	2441 (19.413)	1387 (15.705)	
1.3–3.5	5483 (35.582)	3291 (35.399)	2192 (35.914)	
≥3.5	4543 (46.324)	2778 (45.188)	1765 (48.381)	
Drinking status, *n* (%)				<0.001
Never	1888 (10.077)	1333 (11.350)	555 (7.772)	
Former	2023 (11.504)	1017 (9.798)	1006 (14.594)	
Current	9943 (78.419)	6160 (78.852)	3783 (77.635)	
Smoking status, *n* (%)				<0.001
Never	7587 (54.978)	5103 (58.815)	2484 (48.028)	
Former	3360 (24.654)	1490 (19.237)	1870 (34.468)	
Current	2907 (20.368)	1917 (21.949)	990 (17.504)	
Hypertension, *n* (%)				<0.001
No	8161 (63.888)	5952 (74.123)	2209 (45.344)	
Yes	5693 (36.112)	2558 (25.877)	3135 (54.656)	
DM, *n* (%)				<0.001
No	11,403 (86.839)	7617 (92.813)	3786 (76.016)	
Yes	2451 (13.161)	893 (7.187)	1558 (23.984)	
CVD, *n* (%)				<0.001
No	12,448 (92.544)	8008 (95.605)	4440 (86.997)	
Yes	1406 (7.456)	502 (4.395)	904 (13.003)	

*Note*: Data represent mean (SD) for continuous variables and *n* (%) for categorical variables (percent values were weighted to account for the complex survey design).

Abbreviations: BMI, body mass index; BRI, body roundness index; CVD, cardiovascular disease; DM, diabetes mellitus; OSAS, obstructive sleep apnea syndrome; PIR, poverty‐to‐income ratio.

Among participants diagnosed with OSAS, 795 individuals (14.9%) experienced all‐cause mortality during the follow‐up period (Table 2). Compared to survivors, decedents were significantly older (67.6 ± 0.6 vs. 52.0 ± 0.3 years, *p* < 0.001), had lower BMI (31.9 ± 0.3 vs. 34.0 ± 0.2, *p* < 0.001), and exhibited a greater burden of comorbid conditions, including hypertension (69.5% vs. 53.1%), DM (36.4% vs. 22.7%), and cardiovascular disease (36.3% vs. 10.5%; all *p* < 0.001) (Table 2). All analyses incorporated NHANES sampling weights to account for the complex survey design.

**TABLE 2:** Weighted baseline characteristics of OSAS patients by all‐cause mortality status

Characteristic	Total	No all‐cause mortality	All‐cause mortality	p value
	*N* = 5342	*N* = 4637	*N* = 795	
Age, year, mean (SE)	53.513 (0.339)	52.016 (0.320)	67.620 (0.647)	<0.001
BRI, mean (SE)	6.938 (0.061)	6.944 (0.066)	6.880 (0.100)	0.582
BMI, mean (SE)	33.804 (0.165)	34.005 (0.177)	31.908 (0.302)	<0.001
Gender, *n* (%)				0.015
Female	1266 (23.919)	1125 (24.417)	141 (19.227)	
Male	4076 (76.081)	3422 (75.583)	654 (80.773)	
Race, *n* (%)				0.200
Mexican American	850 (7.663)	790 (8.166)	60 (2.922)	
Non‐Hispanic Black	1176 (10.339)	1002 (10.186)	174 (11.784)	
Non‐Hispanic White	2438 (71.856)	1929 (70.907)	509 (80.804)	
Others	878 (10.142)	826 (10.742)	52 (4.490)	
Marital status, *n* (%)				<0.001
Never married	482 (8.763)	436 (9.066)	46 (5.912)	
Divorced/separated/widowed	1069 (16.653)	835 (15.511)	234 (27.413)	
Married/living with partner	3791 (74.584)	3276 (75.423)	515 (66.675)	
Education level, *n* (%)				0.02
High school	1349 (14.695)	1055 (13.125)	294 (29.497)	
Less than high school	1284 (25.948)	1078 (25.737)	206 (27.938)	
More than high school	2709 (59.357)	2414 (61.138)	295 (42.565)	
PIR, *n* (%)				0.340
<1.3	1386 (15.693)	1146 (14.883)	240 (23.320)	
1.3–3.5	2192 (35.923)	1798 (34.350)	394 (50.748)	
≥3.5	1764 (48.384)	1603 (50.767)	161 (25.933)	
Drinking status, *n* (%)				<0.001
Never	554 (7.757)	456 (7.414)	98 (10.991)	
Former	1005 (14.588)	706 (12.464)	299 (34.605)	
Current	3783 (77.655)	3385 (80.122)	398 (54.404)	
Smoking status, *n* (%)				<0.001
Never	2482 (48.015)	2221 (49.589)	261 (33.177)	
Former	1870 (34.477)	1480 (33.304)	390 (45.532)	
Current	990 (17.508)	846 (17.107)	144 (21.292)	
Hypertension, *n* (%)				<0.001
No	2208 (45.339)	1983 (46.908)	225 (30.548)	
Yes	3134 (54.661)	2564 (53.092)	570 (69.452)	
DM, *n* (%)				<0.001
No	3784 (76.010)	3307 (77.330)	477 (63.564)	
Yes	1558 (23.990)	1240 (22.670)	318 (36.436)	
CVD, *n* (%)				<0.001
No	4438 (86.993)	3949 (89.461)	489 (63.733)	
Yes	904 (13.007)	598 (10.539)	306 (36.267)	

*Note*: Data represent mean (SD) for continuous variables and *n* (%) for categorical variables (percent values were weighted to account for the complex survey design).

Abbreviations: BMI, body mass index; BRI, body roundness index; CVD, cardiovascular disease; DM, diabetes mellitus; OSAS, obstructive sleep apnea syndrome; PIR, poverty‐to‐income ratio.

### 3.2 Correlation Between BRI/BMI and OSAS

The analysis revealed strong, dose‐dependent associations between both adiposity indices (BRI and BMI) and OSAS risk across all models. For BRI, each standard deviation increase conferred progressively higher ORs from Model 1 (crude: OR = 1.846, 95% CI = 1.787–1.906) to fully adjusted Model 3 (OR = 3.060, 95% CI = 2.880–3.251, *p* < 0.001), with quartile analyses demonstrating particularly striking gradients (Q4 vs. Q1: OR = 250.383, 95% CI = 184.018–340.683) (Table 3). Similarly, BMI showed significant per‐SD increments (Model 3: OR = 1.573, 95% CI = 1.535–1.612) and significant‐quartile disparities (Q4: OR = 330.794, 95% CI = 238.468–458.866) (Table 3).

**TABLE 3:** Association between two indicators of obesity and OSAS in weighted multivariable logistic regression.

Characteristic	Model 1		Model 2		Model 3	
	OR (95% CI)	p value	OR (95% CI)	p value	OR (95% CI)	p value
BRI (continues‐per SD)	1.846 (1.787, 1.906)	<0.001	3.033 (2.862, 3.215)	<0.001	3.060 (2.880, 3.251)	<0.001
BRI (quartile)						
Q1	Reference		Reference		Reference	
Q2	5.252 (4.299, 6.416)	<0.001	4.255 (3.432, 5.277)	<0.001	4.019 (3.250, 4.972)	<0.001
Q3	13.355 (10.891, 16.375)	<0.001	19.296 (15.059, 24.723)	<0.001	18.473 (14.335, 23.805)	<0.001
Q4	34.956 (28.540, 42.814)	<0.001	271.860 (200.653, 368.337)	<0.001	250.383 (184.018, 340.683)	<0.001
p for trend		<0.001		<0.001		<0.001
BMI (continues‐per SD)	1.243 (1.229, 1.257)	<0.001	1.573 (1.535, 1.611)	<0.001	1.573 (1.535, 1.612)	<0.001
BMI (quartile)						
Q1	Reference		Reference		Reference	
Q2	4.014 (3.265, 4.935)	<0.001	3.934 (2.938, 5.267)	<0.001	3.843 (2.898, 5.096)	<0.001
Q3	10.560 (8.994, 12.398)	<0.001	18.252 (14.265, 23.355)	<0.001	17.431 (13.685, 22.203)	<0.001
Q4	32.735 (26.664, 40.186)	<0.001	367.018 (264.017, 510.203)	<0.001	330.794 (238.468, 458.866)	<0.001
p for trend		<0.001		<0.001		<0.001

Model 1 (crude model): BRI.

Model 2: age, gender, race, marital status, PIR, education level, drinking status, and smoking status.

Model 3: age, gender, race, marital status, drinking status, smoking status, education level, PIR, hypertension, DM, and CVD.

Abbreviations: BMI, body mass index; BRI, body roundness index; CI, confidence interval; CVD, cardiovascular disease; DM, diabetes mellitus; OR, odds ratio; OSAS, obstructive sleep apnea syndrome; PIR, poverty‐to‐income ratio.

**FIGURE 2:**
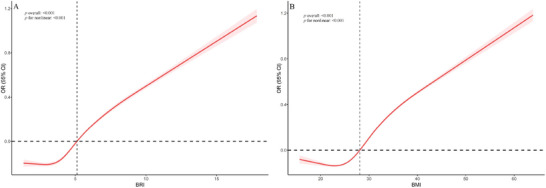
RCS charts representing the relationships of BRI (A) and BMI (B) with OSAS. BRI, body roundness index; BMI, body mass index; OSAS, obstructive sleep apnea syndrome; RCS, restricted cubic spline; OR, odds ratio; CI, confidence interval.

RCS analyses confirmed nonlinear relationships for both indices (*p* for overall <0.001; *p* for nonlinear <0.001), with steeper risk escalation at higher values (Figure 2). The magnitude of association was consistently greater for BRI than BMI across all comparisons, evidenced by higher OR in equivalent models and more pronounced quartile gradients. All trend tests remained statistically significant (*p* < 0.001) after comprehensive adjustment for demographic, socioeconomic, behavioral, and metabolic confounders.

### 3.3 Association Between BRI/BMI and All‐Cause Mortality in OSAS Patients

The analysis demonstrated complex associations between adiposity measures and all‐cause mortality risk in OSAS patients, with differential patterns emerging for BRI and BMI. For BRI, continuous analysis revealed a significant all‐cause mortality association in fully adjusted models (HR = 1.087 per SD, 95% CI = 1.036–1.141, *p* < 0.001), while quartile analysis showed a dose–response relationship (Q4 vs. Q1: HR = 1.423, 95% CI = 1.073–1.887, *p* = 0.014; *p*‐trend < 0.001) (Table 4). BMI exhibited an inverse crude association (HR = 0.964 per SD, 95% CI = 0.949–0.979, *p* < 0.001) that reversed after adjustment (Model 3: HR = 1.021, 95% CI = 1.001–1.040, *p* = 0.038), with quartile analysis showing similar reversal (Q4: HR = 1.378, 95% CI = 0.966–1.964, *p* = 0.037; *p*‐trend = 0.054) (Table 4).

**TABLE 4:** Multivariate Cox regression analysis of two indicators of obesity and all‐cause mortality among individuals with OSAS.

Characteristic	Model 1		Model 2		Model 3	
	HR (95% CI)	p value	HR (95% CI)	p value	HR (95% CI)	p value
All‐cause mortality						
BRI (continues‐per SD)	1.021 (0.987, 1.057)	0.227	1.130 (1.083, 1.180)	<0.001	1.087 (1.036, 1.141)	<0.001
BRI (quartile)						
Q1	Reference		Reference		Reference	
Q2	0.867 (0.661, 1.137)	0.302	0.836 (0.650, 1.075)	0.163	0.834 (0.621, 1.120)	0.227
Q3	1.133 (0.872, 1.473)	0.349	1.147 (0.897, 1.466)	0.274	0.972 (0.741, 1.276)	0.837
Q4	1.115 (0.865, 1.436)	0.401	1.781 (1.364, 2.327)	<0.001	1.423 (1.073, 1.887)	0.014
p for trend		0.026		<0.001		<0.001
BMI (continues‐per SD)	0.964 (0.949, 0.979)	<0.001	1.030 (1.010, 1.050)	0.003	1.021 (1.001, 1.040)	0.038
BMI (quartile)						
Q1	Reference		Reference		Reference	
Q2	1.379 (1.074, 1.771)	0.012	0.951 (0.757, 1.196)	0.669	0.978 (0.762, 1.257)	0.864
Q3	0.839 (0.627, 1.122)	0.237	1.019 (0.782, 1.327)	0.891	0.927 (0.711, 1.208)	0.573
Q4	0.724 (0.529, 0.989)	0.042	1.615 (1.158, 2.252)	0.005	1.378 (0.966, 1.964)	0.037
p for trend		<0.001		0.003		0.054

Model 1 (Crude model): BRI.

Model 2: age, gender, race, marital status, PIR, education level, drinking status, and smoking status.

Model 3: age, gender, race, marital status, drinking status, smoking status, education level, PIR, hypertension, DM, and CVD.

Abbreviations: BMI, body mass index; BRI, body roundness index; CI, confidence interval; CVD, cardiovascular disease; DM, diabetes mellitus; HR, hazard ratio; OSAS, obstructive sleep apnea syndrome; PIR, poverty‐to‐income ratio.

**FIGURE 3:**
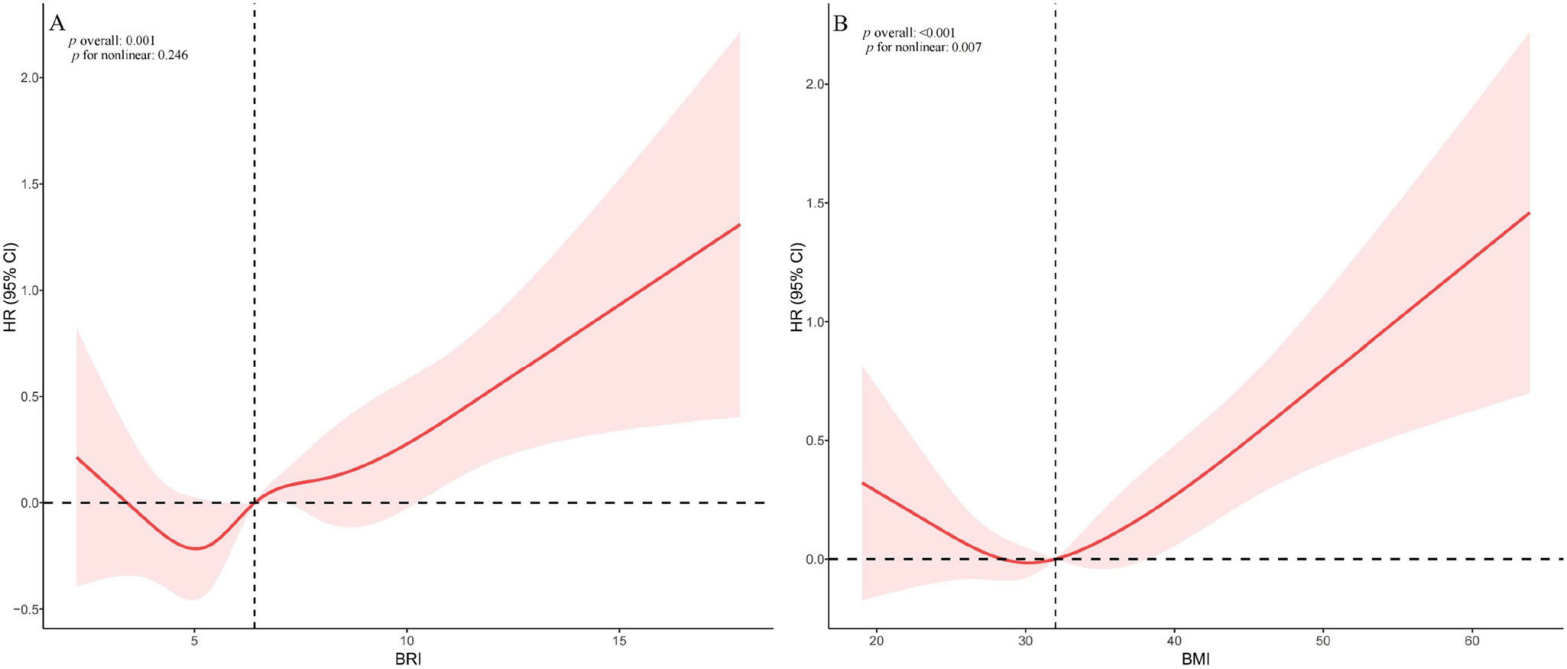
RCS charts representing the relationships of BRI (A) and BMI (B) with all‐cause mortality in OSAS individuals. BRI, body roundness index; BMI, body mass index; OSAS, obstructive sleep apnea syndrome; RCS, restricted cubic spline; OR, odds ratio; CI, confidence interval.

**FIGURE 4:**
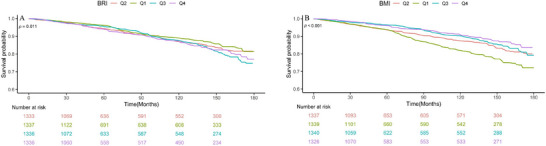
Kaplan–Meier curves illustrate survival probabilities across BRI (A) and BMI (B) quartiles (Q1–Q4) during follow‐up in OSAS patients. The *Y*‐axis shows survival probability, while the *X*‐axis represents follow‐up time. BRI, body roundness index; BMI, body mass index; OSAS, obstructive sleep apnea syndrome.

RCS analysis further confirmed the presence of a linear relationship between BRI and all‐cause mortality (*p* for overall = 0.001; *p* for nonlinear = 0.246), while the association between BMI and all‐cause mortality showed a statistically significant nonlinear pattern (*p* for overall <0.001; *p* for nonlinear = 0.007) (Figure 3). Kaplan–Meier curves substantiated these findings, revealing significant survival disparities across BRI quartiles (*p* < 0.001), with Q4 showing poorest outcomes, while BMI quartiles showed attenuated but still significant stratification (*p* = 0.011) (Figure 4).

### 3.4 Subgroup Analysis

Subgroup analyses demonstrated significant variations in adiposity–OSAS associations across populations. Both BRI and BMI exhibited stronger OSAS correlations in younger patients (BRI OR = 2.63, 95% CI = 2.48–2.78; BMI OR = 8.33, 95% CI = 7.92–8.74) and females (BRI OR = 4.33, 95% CI = 4.11–4.55; BMI OR = 12.61, 95% CI = 11.96–13.27), with notable age (*p*‐interaction < 0.001) and gender (*p*‐interaction < 0.001) interactions (Tables S3 and S4). For mortality risk in OSAS patients, BRI showed significant effect modification by education level (*p*‐interaction = 0.017) with strongest association in higher‐educated groups (HR = 1.075, 95% CI = 1.022–1.130), while BMI demonstrated significant variation by marital status (*p*‐interaction = 0.007) with most pronounced protective effect in divorced/separated/widowed individuals (HR = 0.936, 95% CI = 0.912–0.960) (Tables S5 and S6). No significant interaction was observed for smoking status with BRI–mortality association (*p*‐interaction = 0.646), whereas BMI showed an additional significant interaction by education level (*p*‐interaction = 0.021) (Tables S5 and S6). Both indices maintained consistent associations across racial/ethnic groups (BRI *p*‐interaction = 0.832; BMI *p*‐interaction = 0.789) (Tables S5 and S6), with all analyses incorporating full adjustment for clinical covariates and NHANES survey weights.

### 3.5 Comparative Performance of BRI and BMI for in Predicting OSAS and Mortality Risk

**FIGURE 5:**
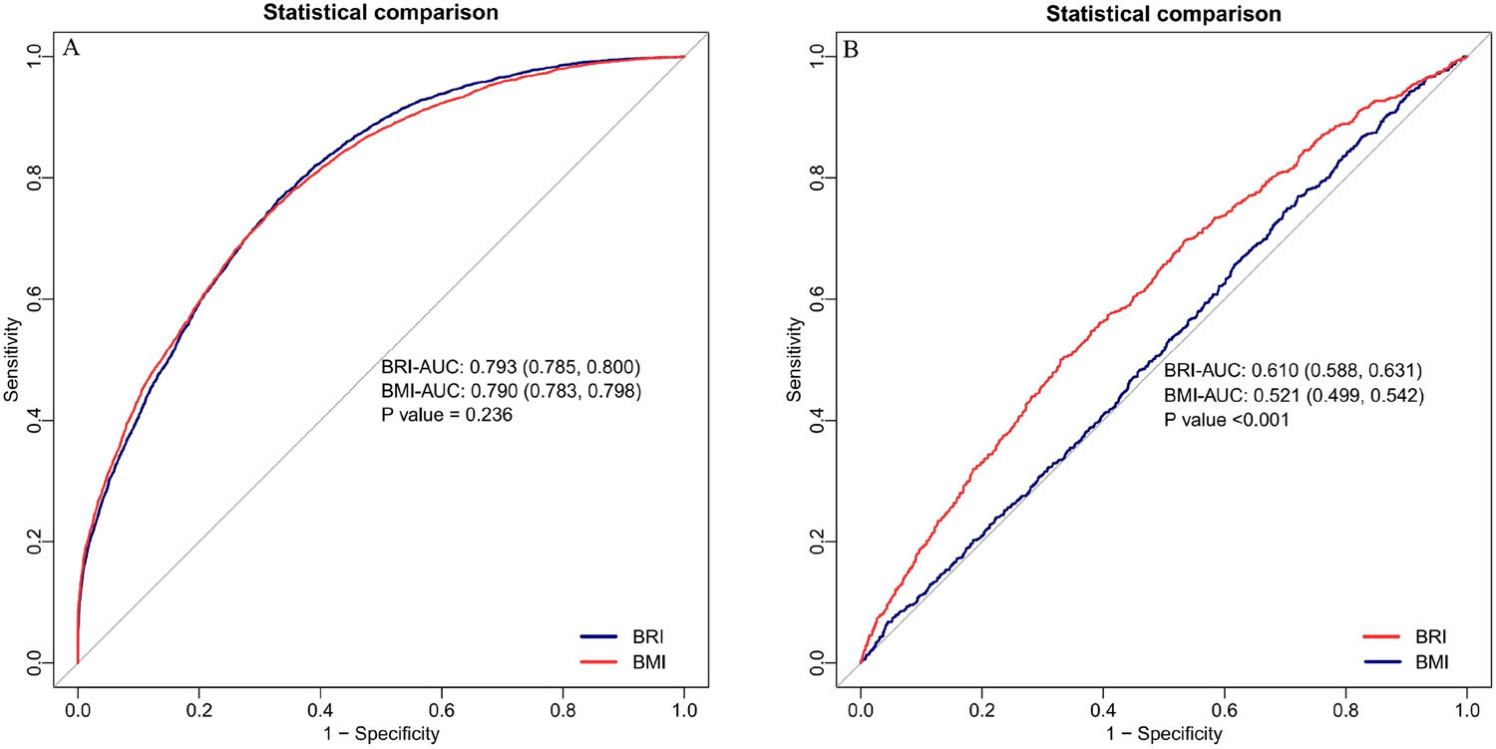
Comparative predictive performance of BRI and BMI for OSAS risk (A) and all‐cause mortality in individuals with OSAS (B). Panel A displays curves comparing the performance of the BRI and BMI in predicting OSAS. Panel B presents ROC curves for BRI and BMI in predicting all‐cause mortality among individuals with OSAS. BRI, body roundness index; BMI, body mass index; OSAS, obstructive sleep apnea syndrome; AUC, area under the curve; ROC, receiver operating characteristic.

ROC analyses demonstrated differential predictive performance of BRI and BMI for OSAS risk versus mortality outcomes. For OSAS prediction, BRI showed comparable discriminative ability to BMI (AUC = 0.793, 95% CI: 0.785–0.800 vs. AUC = 0.790, 95% CI: 0.783–0.798; *p* = 0.236) (Figure 5), with overlapping CIs suggesting no statistically significant difference. In contrast, for all‐cause mortality prediction among OSAS patients, BRI exhibited superior performance (AUC = 0.610, 95% CI: 0.588–0.631) (Figure 5) compared to BMI (AUC = 0.521, 95% CI: 0.499–0.542; *p* < 0.001) (Figure 5). These findings suggest BRI may offer particular advantages over BMI in mortality risk assessment among OSAS patients, despite their comparable performance for initial disease prediction.

### 3.6 IDI and NRI Assessment of BRI Versus BMI for Mortality Risk Stratification in OSAS

The NRI and IDI indices were calculated to compare how well the BRI and BMI predict mortality risk in patients with OSAS. The model using BMI indicated a categorical NRI of 0.164 (95% CI: 0.145–0.183), a continuous NRI of 0.356 (95% CI: 0.321–0.392), and an IDI of 0.034 (95% CI: 0.031–0.037), all with *p* < 0.001. In comparison, the BRI‐based model achieved slightly greater values, with a categorical NRI of 0.171 (95% CI: 0.151–0.190), a continuous NRI of 0.358 (95% CI: 0.323–0.394), and an IDI of 0.036 (95% CI: 0.033–0.039), all with *p* < 0.001. Despite the modest magnitude of improvement, the consistent increases across all reclassification metrics show that BRI offers a measurable yet limited enhancement in mortality risk discrimination relative to BMI.

## 4 Discussion

This large‐scale, nationally representative study demonstrates that while both BRI and BMI exhibit strong associations with OSAS, BRI outperforms BMI in predicting all‐cause mortality among individuals with OSAS. RCS analysis further confirmed the presence of a linear relationship between BRI and all‐cause mortality (*p* for overall = 0.001; *p* for nonlinear = 0.246), while the association between BMI and all‐cause mortality showed a statistically significant nonlinear pattern (*p* for overall <0.001; *p* for nonlinear = 0.007). For OSAS prediction, both indices had comparable discriminative capacity (AUC: BRI = 0.793 vs. BMI = 0.790, *p* = 0.236), but BRI's superior performance in mortality risk stratification (AUC = 0.610 vs. BMI = 0.521, *p* < 0.001) underscores its clinical utility as a more sensitive adiposity metric, particularly for visceral fat‐related prognostic assessment. These findings suggest that BRI may refine risk stratification beyond conventional BMI in OSAS management.

Our study provides novel insights into the differential clinical utility of adiposity indices by demonstrating that BRI, unlike BMI, has superior prognostic value for mortality in OSAS patients, challenging conventional anthropometric paradigms. The robust association between BRI and all‐cause mortality (HR = 1.423 for Q4 vs. Q1) extends beyond previous reports in metabolic syndrome populations by specifically implicating visceral adiposity in OSAS‐related mortality pathways (Chen et al. 2025). This observation gains mechanistic plausibility from recent work by Lee et al. (2012) showing that central obesity potentiates oxidative stress in OSAS patients and from Sacramento et al.’s (2023) demonstration of gender‐specific respiratory impacts of abdominal adiposity. Importantly, our study clarifies the controversial obesity–mortality relationship in OSAS by demonstrating that BMI's apparent protective association (crude HR = 0.964 per SD) became marginally harmful after full adjustment (adjusted HR = 1.021). Subgroup analyses revealed notable heterogeneity in the relationship between adiposity indices and OSAS across educational strata. BRI maintained a consistently strong association with OSAS across all education levels (all *p* < 0.001); however, its predictive utility for all‐cause mortality was most pronounced among individuals with higher educational attainment (HR = 1.075; 95% CI: 1.022–1.130; *p* = 0.005). This observed education‐related gradient (*p*‐interaction = 0.101) may reflect more effective healthcare engagement or residual socioeconomic confounding, aligning with previous findings on education‐mediated disparities in health outcomes (Suiter and Meadows 2023; Murakami et al. 2023). These results suggest that BRI may hold particular prognostic value in health‐literate populations, where the precision of risk stratification and communication is critical. The striking gender disparity in OSAS risk (women: BRI OR = 4.331 vs. men: OR = 2.430) is significant, demonstrating that this dimorphism persists even after accounting for mortality outcomes. This pattern was even more pronounced for BMI, with markedly stronger associations observed in women (OR = 12.612) compared to men (OR = 6.595), reinforcing the need for sex‐specific adiposity thresholds in the assessment and management of obesity‐related respiratory disorders (Petermann‐Rocha et al. 2020; Kisiel et al. 2023; Wei et al. 2025). Unexpectedly, we found no racial/ethnic modification of BRI–mortality associations, contrasting with Okobi et al.’s (2023) and Ashraf et al.’s (2024) reports of ethnic variability in adiposity–mortality relationships. This discrepancy may reflect our focus on OSAS‐specific mortality rather than general cardiometabolic outcomes, suggesting that visceral adiposity's pathophysiological impact may be more uniform across ethnicities in the context of sleep‐disordered breathing. Despite the ORs being significantly higher for the top quartiles of BRI and BMI, the CIs expanded proportionately, and the direction of the associations was uniform across all models. The pattern reflects a stable statistical framework with limited heterogeneity, rather than indicating model overfitting or instability. These pronounced associations likely reflect a steep, nonlinear rise in OSAS risk with greater adiposity, especially when central and visceral fat are extreme, as shown in prior population‐based and mechanistic research (Kariuki et al. 2022; Schwartz et al. 2008; Jeong et al. 2017). Nonetheless, these findings should be considered carefully, given the potential influence of sparse data in the upper exposure distribution and the inherent limitations of categorization. Although IDI and NRI improvements were limited, this does not necessarily impact the practical or clinical significance of BRI in mortality risk evaluation for OSAS patients. Several factors could contribute to the modest incremental indices. First and foremost, IDI and NRI are sensitive to the prevalence of the outcome and the model's calibration; when the baseline model is already performing well, even meaningful clinical improvements may appear numerically small. In the second instance, both metrics focus on statistical rather than clinical enhancement, and even a minimal change can be key for identifying high‐risk subgroups. Third, given the strong correlation between BMI and BRI, both of which represent general adiposity, the measurable incremental gain might be smaller, even though BRI more accurately captures the metabolic risk associated with visceral fat. Hence, although the quantitative reclassification metrics were not strong, the consistent effects and theoretical advantages of BRI suggest it could still provide supplementary value for mortality risk stratification in OSAS populations.

The shift in BMI's association with mortality, from a protective crude estimate to a harmful adjusted one, is consistent with previous findings of the “obesity paradox.” First, reverse causality is plausible: pre‐existing disease and catabolic states can induce unintentional weight loss, shifting individuals with high underlying risk into lower BMI categories and producing spurious survival advantages for higher BMI strata in unadjusted models (Dramé and Godaert 2023). Second, frailty and sarcopenia (low lean mass) are often incompletely captured in large surveys; because BMI conflates fat and lean compartments, low BMI may reflect disease‐related wasting rather than intrinsically lower risk, while higher BMI can proxy preserved reserves in some clinical contexts (Koon‐Yee Lee et al. 2021). In the presence of incomplete confounding control, both mechanisms can distort crude associations and diminish or invert true connections. Crucially, these biases may be less noticeable in indices of adiposity that show fat distribution, especially visceral adiposity. Growing evidence shows that central/visceral fat better reflects cardiometabolic risk and mortality than total mass, while BMI remains insensitive to fat distribution and is affected by lean mass (Zhang et al. 2024). Recent research involving cohorts and methodologies shows that BRI and related visceral fat surrogates (such as VAI and METS‐VF) predict mortality and adverse outcomes more reliably than BMI, which supports their greater physiological specificity (Xue et al. 2025; Xie et al. 2025; Zeng et al. 2025). In our data, the stronger and more linear association between BRI and mortality in OSAS may reflect its closer linkage to pathophysiological mechanisms, including upper airway collapsibility, impaired ventilatory control, systemic inflammation, and overall cardiometabolic burden, compared with BMI. Finally, in cardiorespiratory situations, the obesity paradox is frequently less pronounced after adjusting for disease severity, cardiorespiratory fitness, or sleep‐related breathing disorders; recent studies indicate that the perceived survival advantage of a higher BMI during acute cardiovascular incidents might be partially due to the presence of sleep apnea phenotypes and biases in selection or measurement (Donovan and Au 2022; Sharafkhaneh et al. 2023). Hence, although the quantitative reclassification indices were not strong, the consistent effect direction and theoretical reasoning behind BRI indicate it could still be beneficial for risk stratification in OSAS.

OSAS pathogenesis might be influenced by the BRI via multiple related mechanisms involving visceral adiposity. First, the mechanical load from abdominal fat accumulation, indicated by BRI, diminishes lung volumes and tracheal traction, which in turn raises the collapsibility of the upper airway during sleep (Messineo et al. 2024; Abourjeili et al. 2024). Second, visceral fat tissue emits pro‐inflammatory cytokines like IL‐6 and TNF‐α, which hinder the neuromuscular regulation of pharyngeal dilator muscles (Palma et al. 2022; Silva‐Reis et al. 2024). Third, metabolic dysfunctions tied to BRI, like insulin resistance connected to intermittent hypoxia and REM sleep disturbances, result in breathing patterns that are unstable (Mangas‐Moro et al. 2024). Notably, the geometric model underlying BRI better captures fat distribution patterns that compress the thorax and diaphragm compared to BMI, as demonstrated by imaging studies linking android obesity with reduced pharyngeal lumen area (Alfuriji et al. 2024; Xia et al. 2025; Sutherland et al. 2012). These mechanisms collectively explain why BRI shows stronger associations with OSAS severity than BMI in clinical studies.

Although this cohort is nationally representative and employs rigorous statistical techniques, there are several limitations to consider. First, the identification of OSAS was done through an adapted multivariable apnea prediction index rather than the standard polysomnography. Previous studies validating this instrument have found high sensitivity (about 88%), but the relatively low specificity (around 55%) may have led to some misclassification of outcomes. Misclassification of this nature is likely nondifferential concerning adiposity indices, which means any bias would likely push the true associations toward the null rather than exaggerate them. Hence, although the high ORs observed in our analysis may seem substantial, they probably reflect careful estimates of the true associations rather than being inflated by misclassification. Second, even with comprehensive covariate adjustments, residual confounding could remain due to unmeasured variables such as exercise habits, nutrition, sleep structure, or environmental factors. Third, even though BRI provides benefits over BMI in estimating central adiposity, it is determined solely by WC and height and lacks validation against imaging‐derived measures of visceral fat in this cohort. Fourth, the cross‐sectional nature of the NHANES data on OSAS prevents the establishment of temporal or causal relationships between adiposity indices (BRI and BMI) and the risk of OSAS. Although the analysis of mortality outcomes was done prospectively, the observational framework of NHANES might still encounter residual confounding and potential reverse causation. Thus, although our findings show notable connections, they should be viewed as correlational and not causal. Lastly, while subgroup analyses offered insights into effect modification by education, sex, and marital status, several interaction effects were marginal and should be interpreted cautiously due to the potential for type I error arising from multiple testing. Future studies need to verify the anatomical accuracy of the BRI by comparing it with imaging techniques like computed tomography or MRI for measuring visceral fat. More research should be conducted to evaluate the clinical effectiveness of BRI‐based risk stratification strategies in real‐world settings to ascertain whether BRI improves the prediction and management of outcomes related to OSA.

## 5 Conclusion

In this cohort that represents the nation, both the BRI and BMI were significantly correlated with the risk of developing symptoms of OSAS. Nonetheless, BRI exhibited a stronger and more reliable relationship. Furthermore, BRI was more effective than BMI in forecasting all‐cause mortality in people with OSAS, as shown by higher HRs and better discriminatory ability. BRI, which is based on geometric measurements, might be a more dependable tool for risk evaluation in sleep‐disordered breathing and associated mortality because it better captures visceral adiposity. Integrating BRI into regular clinical procedures might improve the early detection of individuals at high risk.

## Author Contributions

C.X.D. and C.X.L. were responsible for the conception and design of the study. C.X.D. and L.H.W. executed the data collection and analysis. W.Z.J., L.H.W., and Z.M.H. designed the clinical protocols. The initial draft of the manuscript was created by C.X.D. and W.Z.J. The final version of the manuscript was approved for publication by all authors after a critical review for intellectual content.

## Funding

The authors have nothing to report.

## Ethics Statement

The Institutional Review Board of the National Center for Health Statistics, affiliated with the CDC, approved the NHANES study protocols ethically. Prior to participating in this study, written informed consent was collected from each participant. This investigation followed the Declaration of Helsinki guidelines and employed de‐identified, publicly available NHANES data.

## Conflicts of Interest

The authors declare no conflicts of interest.

## Supporting information




**Supplementary Material**: brb371109‐sup‐0001‐SuppMat.docx

## Data Availability

This investigation uses data from the NHANES (https://wwwn.cdc.gov/nchs/nhanes/default.aspx), which is publicly accessible.
