# Supplementary material for: Body Roundness Index Versus Body Mass Index: Differential Associations With Obstructive Sleep Apnea Syndrome and All‐Cause Mortality in US Adults Aged 20 Years and Older
Source: Brain Behav. 2025 Dec 2;15(12):e71109. doi: 10.1002/brb3.71109 (PMC12672924; doi:10.1002/brb3.71109)
Supplement: Supplementary file 1 — Supplementary Material: brb371109‐sup‐0001‐SuppMat.docx [file BRB3-15-e71109-s001.docx]

**Supplementary Table 1** Variance inflation factor analysis of clinical covariates (BRI-OSAS)

| **Characteristics** | **VIF** |
| --- | --- |
| **Age, (years)** | 1,8 |
| **Gender, n (%)** | 1.1 |
| **Race, n (%)** | 1.0 |
| **Marital_status, n (%)** | 1.3 |
| **Education_level, n (%)** | 1.4 |
| **PIR, n (%)** | 1.2 |
| **Drinking_status, n (%)** | 1.1 |
| **Smoking_status, n (%)** | 1.1 |
| **Hypertension, n (%)** | 1.2 |
| **DM, n (%)** | 1.1 |
| **CVD, n (%)** | 1.1 |

**Abbreviation:** BRI, body roundness index; BMI, body mass index; OSAS, obstructive sleep apnea syndrome; PIR, poverty-to-income ratio; DM, diabetes mellitus; CVD, cardiovascular disease

**Supplementary Table 2** Variance inflation factor analysis of clinical covariatesVariance inflation factor analysis of clinical covariates (BMI-OSAS)

| **Characteristics** | **VIF** |
| --- | --- |
| **Age, (years)** | 1.9 |
| **Gender, n (%)** | 1.1 |
| **Race, n (%)** | 1.3 |
| **Marital_status, n (%)** | 1.3 |
| **Education_level, n (%)** | 1.4 |
| **PIR, n (%)** | 1.3 |
| **Drinking_status, n (%)** | 1.2 |
| **Smoking_status, n (%)** | 1.3 |
| **Hypertension, n (%)** | 1.3 |
| **DM, n (%)** | 1.2 |
| **CVD, n (%)** | 1.1 |

**Abbreviation:** BMI, body mass index; OSAS, obstructive sleep apnea syndrome; PIR, poverty-to-income ratio; DM, diabetes mellitus; CVD, cardiovascular disease

**Supplementary Table 3** The relationship between BRI and OSAS in various subgroups

| **Characteristic** | **OR (95%CI)** | **P value** | ***P* for interaction** |
| --- | --- | --- | --- |
| **Age** |  |  | < 0.001 |
| <60 | 2.627 (2.475,2.779) | <0.001 |  |
| >=60 | 1.892 (1.740,2.044) | <0.001 |  |
| **Gender** |  |  | < 0.001 |
| Female | 4.331 (4.108,4.554) | <0.001 |  |
| Male | 2.430 (2.333,2.527) | <0.001 |  |
| **Race, n (%)** |  |  | < 0.001 |
| Mexican american | 2.883 (2.642,3.123) | <0.001 |  |
| Non-hispanic black | 2.503 (2.352,2.655) | <0.001 |  |
| Non-hispanic white | 2.224 (1.946,2.503) | <0.001 |  |
| Others | 2.178 (1.909,2.447) | <0.001 |  |
| **Marital_status, n (%)** |  |  | < 0.001 |
| Never married | 3.845 (3.396,4.293) | <0.001 |  |
| Divorced/Separated/Widowed | 2.577 (2.343,2.810) | <0.001 |  |
| Married/Living with partner | 2.227 (2.098,2.357) | <0.001 |  |
| **Education_level, n (%)** |  |  | 0.009 |
| High school | 2.114 (1.906,2.323) | <0.001 |  |
| Less than High school | 2.530 (2.292,2.769) | <0.001 |  |
| More than High school | 2.534 (2.381,2.686) | <0.001 |  |
| **PIR, n (%)** |  |  | 0.013 |
| <1.3 | 2.823 (2.499,3.148) | <0.001 |  |
| 1.3-3.5 | 2.575 (2.374,2.777) | <0.001 |  |
| ≥3.5 | 2.334 (2.170,2.498) | <0.001 |  |
| **Drinking_status, n (%)** |  |  | 0.269 |
| Never | 2.658 (2.353,2.964) | <0.001 |  |
| Former | 2.325 (2.068,2.582) | <0.001 |  |
| Current | 2.480 (2.331,2.630) | <0.001 |  |
| **Smoking_status, n (%)** |  |  | < 0.001 |
| Never | 2.647 (2.508,2.787) | <0.001 |  |
| Former | 2.298 (2.106,2.490) | <0.001 |  |
| Current | 2.254 (2.005,2.502) | <0.001 |  |
| **Hypertension, n (%)** |  |  | 0.619 |
| No | 2.280 (2.105,2.456) | <0.001 |  |
| Yes | 2.231 (2.084,2.377) | <0.001 |  |
| **DM, n (%)** |  |  | 0.089 |
| No | 2.289 (2.157,2.421) | <0.001 |  |
| Yes | 2.078 (1.841,2.315) | <0.001 |  |
| **CVD, n (%)** |  |  | 0.001 |
| No | 2.478 (2.343,2.613) | <0.001 |  |
| Yes | 1.956 (1.645,2.268) | <0.001 |  |

Mean (sd) for continuous; n (%) for categorical (Percent values were weighted to account for the complex survey design.)

**Abbreviation:** BRI, body roundness index; BMI, body mass index; OSAS, obstructive sleep apnea syndrome; PIR, poverty-to-income ratio; DM, diabetes mellitus; CVD, cardiovascular disease

**Supplementary Table 4** The relationship between BMI and OSAS in various subgroups

| **Characteristic** | **OR (95%CI)** | **P value** | ***P* for interaction** |
| --- | --- | --- | --- |
| **Age** |  |  | < 0.001 |
| <60 | 8.331 (7.917,8.744) | <0.001 |  |
| >=60 | 6.128 (5.687,6.569) | <0.001 |  |
| **Gender** |  |  | < 0.001 |
| Female | 12.612 (11.958,13.266) | <0.001 |  |
| Male | 6.595 (6.304,6.886) | <0.001 |  |
| **Race, n (%)** |  |  | < 0.001 |
| Mexican american | 8.894 (8.187,9.601) | <0.001 |  |
| Non-hispanic black | 7.382 (7.004,7.761) | <0.001 |  |
| Non-hispanic white | 6.763 (5.976,7.551) | <0.001 |  |
| Others | 6.673 (5.882,7.463) | <0.001 |  |
| **Marital_status, n (%)** |  |  | < 0.001 |
| Never married | 11.599(10.362,12.837) | <0.001 |  |
| Divorced/Separated/Widowed | 7.955(7.294,8.616) | <0.001 |  |
| Married/Living with partner | 6.713(6.373,7.052) | <0.001 |  |
| **Education_level, n (%)** |  |  | 0.006 |
| High school | 6.391(5.744,7.039) | <0.001 |  |
| Less than High school | 7.587(6.987,8.187) | <0.001 |  |
| More than High school | 7.558(7.160,7.956) | <0.001 |  |
| **PIR, n (%)** |  |  | 0.01 |
| <1.3 | 8.502 (7.574,9.429) | <0.001 |  |
| 1.3-3.5 | 7.430 (6.914,7.946) | <0.001 |  |
| ≥3.5 | 7.076 (6.656,7.496) | <0.001 |  |
| **Drinking_status, n (%)** |  |  | 0.325 |
| Never | 7.955 (7.055,8.855) | <0.001 |  |
| Former | 7.002 (6.149,7.855) | <0.001 |  |
| Current | 7.397 (7.004,7.790) | <0.001 |  |
| **Smoking_status, n (%)** |  |  | < 0.001 |
| Never | 8.012 (7.608,8.416) | <0.001 |  |
| Former | 6.571 (6.073,7.068) | <0.001 |  |
| Current | 7.206 (6.475,7.937) | <0.001 |  |
| **Hypertension, n (%)** |  |  | 0.514 |
| No | 6.974 (6.476,7.471) | <0.001 |  |
| Yes | 7.173 (6.768,7.579) | <0.001 |  |
| **DM, n (%)** |  |  | 0.723 |
| No | 7.015 (6.649,7.381) | <0.001 |  |
| Yes | 6.887(6.219,7.554) | <0.001 |  |
| **CVD, n (%)** |  |  | 0.007 |
| No | 7.516 (7.163,7.870) | <0.001 |  |
| Yes | 6.468 (5.700,7.236) | <0.001 |  |

Mean (sd) for continuous; n (%) for categorical (Percent values were weighted to account for the complex survey design.)

**Abbreviation:** BMI, body mass index; OSAS, obstructive sleep apnea syndrome; PIR, poverty-to-income ratio; DM, diabetes mellitus; CVD, cardiovascular disease

**Supplementary Table 5** Subgroup analysis of the association between BRI and all-cause mortality in patients with OSAS

| **Characteristic** | **OR (95%CI)** | **P value** | ***P* for interaction** |
| --- | --- | --- | --- |
| **Age** |  |  | 0.939 |
| <60 | 1.038 (0.996, 1.082) | 0.076 |  |
| >=60 | 1.033(0.950,1.123) | 0.449 |  |
| **Gender** |  |  | 0.055 |
| Female | 1.079 (1.032, 1.128) | <0.001 |  |
| Male | 0.957(0.862,1.063) | 0.416 |  |
| **Race, n (%)** |  |  | 0.832 |
| Mexican american | 1.053 (0.963, 1.151) | 0.259 |  |
| Non-hispanic black | 1.001 (0.940, 1.067) | 0.965 |  |
| Non-hispanic white | 1.023 (0.976, 1.072) | 0.347 |  |
| Others | 1.037 (0.950, 1.132) | 0.416 |  |
| **Marital_status, n (%)** |  |  | 0.017 |
| Never married | 1.008(0.939,1.081) | 0.834 |  |
| Divorced/Separated/Widowed | 0.962(0.903,1.023) | 0.218 |  |
| Married/Living with partner | 1.035(0.990,1.082) | 0.125 |  |
| **Education_level, n (%)** |  |  | 0.101 |
| High school | 0.995 (0.925, 1.070) | 0.882 |  |
| Less than High school | 0.949 (0.896, 1.006) | 0.077 |  |
| More than High school | 1.075 (1.022, 1.130) | 0.005 |  |
| **PIR, n (%)** |  |  | 0.383 |
| <1.3 | 1.002 (0.954, 1.052) | 0.952 |  |
| 1.3-3.5 | 0.974 (0.927, 1.023) | 0.296 |  |
| ≥3.5 | 1.038 (0.944, 1.141) | 0.445 |  |
| **Drinking_status, n (%)** |  |  | 0.742 |
| Never | 0.957(0.849, 1.077) | 0.463 |  |
| Former | 0.998 (0.935, 1.065) | 0.949 |  |
| Current | 1.008 (0.956, 1.062) | 0.778 |  |
| **Smoking_status, n (%)** |  |  | 0.646 |
| Never | 1.006 (0.938, 1.080) | 0.860 |  |
| Former | 1.048 (1.003, 1.096) | 0.037 |  |
| Current | 1.054 (0.945, 1.177) | 0.345 |  |
| **Hypertension, n (%)** |  |  | 0.656 |
| No | 0.983 (0.923 ,1.047) | 0.592 |  |
| Yes | 0.999 (0.956, 1.044) | 0.972 |  |
| **DM, n (%)** |  |  | 0.54 |
| No | 0.993 (0.952, 1.035) | 0.733 |  |
| Yes | 0.966 (0.891, 1.048) | 0.404 |  |
| **CVD, n (%)** |  |  | 0.636 |
| No | 1.009 (0.956, 1.065) | 0.734 |  |
| Yes | 0.984 (0.911, 1.063) | 0.683 |  |

Mean (sd) for continuous; n (%) for categorical (Percent values were weighted to account for the complex survey design.)

**Abbreviation:** BRI, body roundness index; BMI, body mass index; OSAS, obstructive sleep apnea syndrome; PIR, poverty-to-income ratio; DM, diabetes mellitus; CVD, cardiovascular disease

**Supplementary Table 6** Subgroup analysis of the association between BMI and all-cause mortality in patients with OSAS

| **Characteristic** | **OR (95%CI)** | **P value** | ***P* for interaction** |
| --- | --- | --- | --- |
| **Age** |  |  | 0.888 |
| <60 | 0.990 (0.973, 1.006) | 0.226 |  |
| >=60 | 0.992 (0.958, 1.027) | 0.645 |  |
| **Gender** |  |  | 0.65 |
| Female | 0.965 (0.946, 0.985) | <0.001 |  |
| Male | 0.952 (0.911, 0.995) | 0.030 |  |
| **Race, n (%)** |  |  | 0.789 |
| Mexican american | 0.979 (0.935, 1.026) | 0.381 |  |
| Non-hispanic black | 0.967 (0.940, 0.995) | 0.021 |  |
| Non-hispanic white | 0.960 (0.940, 0.980) | <0.001 |  |
| Others | 0.976 (0.935, 1.020) | 0.281 |  |
| **Marital_status, n (%)** |  |  | 0.007 |
| Never married | 0.991 (0.962, 1.021) | 0.559 |  |
| Divorced/Separated/Widowed | 0.936 (0.912, 0.960) | <0.001 |  |
| Married/Living with partner | 0.966 (0.948, 0.985) | <0.001 |  |
| **Education_level, n (%)** |  |  | 0.021 |
| High school | 0.957 (0.932, 0.982) | <0.001 |  |
| Less than High school | 0.936 (0.914, 0.958) | <0.001 |  |
| More than High school | 0.988 (0.965, 1.011) | 0.298 |  |
| **PIR, n (%)** |  |  | 0.318 |
| <1.3 | 0.975 (0.955, 0.995) | 0.013 |  |
| 1.3-3.5 | 0.947 (0.925, 0.970) | <0.001 |  |
| ≥3.5 | 0.964 (0.922, 1.008) | 0.105 |  |
| **Drinking_status, n (%)** |  |  | 0.536 |
| Never | 0.937 (0.897, 0.978) | 0.003 |  |
| Former | 0.968 (0.938, 0.998) | 0.039 |  |
| Current | 0.956 (0.933, 0.981) | <0.001 |  |
| **Smoking_status, n (%)** |  |  | 0.65 |
| Never | 0.962 (0.928, 0.997) | 0.035 |  |
| Former | 0.965 (0.942, 0.988) | 0.004 |  |
| Current | 0.986 (0.946, 1.027) | 0.492 |  |
| **Hypertension, n (%)** |  |  | 0.123 |
| No | 0.938 (0.911, 0.965) | <0.001 |  |
| Yes | 0.962 (0.946, 0.978) | <0.001 |  |
| **DM, n (%)** |  |  | 0.319 |
| No | 0.942 (0.921, 0.963) | <0.001 |  |
| Yes | 0.963 (0.930, 0.996) | 0.030 |  |
| **CVD, n (%)** |  |  | 0.88 |
| No | 0.963 (0.939, 0.987) | 0.003 |  |
| Yes | 0.967 (0.932, 1.003) | 0.071 |  |

Mean (sd) for continuous; n (%) for categorical (Percent values were weighted to account for the complex survey design.)

**Abbreviation:** BRI, body roundness index; BMI, body mass index; OSAS, obstructive sleep apnea syndrome; PIR, poverty-to-income ratio; DM, diabetes mellitus; CVD, cardiovascular disease
